# Analysis of Water-Based Polyurethane Properties in the Ballistic Behavior of Ultra-High Molecular Weight Polyethylene Fiber Composites

**DOI:** 10.3390/polym17070837

**Published:** 2025-03-21

**Authors:** Shuhao Yang, Shumao Zhai, Mingxing Piao, Xiao Wang, Haofei Shi, Chaolong Li

**Affiliations:** 1 School of Materials Science and Engineering, Chongqing Jiaotong University, Chongqing 400074, China; yangshuhao@cigit.ac.cn; 2 Chongqing Institute of Green and Intelligent Technology, Chinese Academy of Sciences, Chongqing 400714, China; zhaishumao@cigit.ac.cn (S.Z.); piaomingxing@cigit.ac.cn (M.P.); wangxiao@cigit.ac.cn (X.W.); shihaofei@cigit.ac.cn (H.S.); 3 Chongqing School, University of Chinese Academy of Sciences, Chongqing 400714, China

**Keywords:** water-based polyurethane, hard segment content, ultra-high molecular weight polyethylene, fiber-reinforced polymer composites, ballistic performance

## Abstract

The ballistic performance of fiber-reinforced polymer composites (FRPC) is influenced by the adhesive’s mechanical properties, such as stiffness, toughness, and energy dissipation. However, the specific contributions of these properties remain unclear. This study explores how varying the hard segment (HS) content in water-based polyurethane (WPU) impacts the thermal, mechanical, and ballistic performance of FRPCs. By increasing HS content, the storage modulus and tensile strength of WPU improved, while elongation at break decreased, transitioning the adhesive from soft and ductile to rigid and brittle. Quasi-static tests, ballistic experiments, and SEM analysis were conducted on UHMWPE fiber-reinforced WPU-HS% composites. Results reveal that adhesives with high hardness and modulus hinder fiber deformation, reducing energy dissipation and causing severe delamination, which diminishes ballistic performance. Conversely, soft and ductile adhesives allow deformation alongside fibers during bullet impact, suppress delamination, and absorb more kinetic energy while transferring load. Among the tested formulations, WPU with 45% HS content exhibited the best balance of mechanical properties, achieving the most significant improvement in ballistic performance by enhancing energy absorption and minimizing damage. This study establishes a clear relationship between WPU properties and composite protective behavior, providing insights for designing high-performance ballistic materials.

## 1. Introduction

Nowadays, on the battlefield, soldiers face multiple threats such as bullets, high-velocity fragments, and explosive shock waves. The primary materials used for individual ballistic protection are high-hardness ceramics and fiber-reinforced polymer composites (FRPC) [1,2]. Compared to ceramic-based ballistic composites, fiber-reinforced adhesive ballistic composites (FRPCs) offer several advantages, including lightweight, excellent flexibility, high hardness, high strength, superior energy dissipation and absorption capabilities, enhanced resistance to repeated impacts, and overall better ballistic performance [3,4,5,6,7]. Fiber-reinforced adhesive composites are composed of high-performance fibers and an adhesive matrix. The fibers provide reinforcement, while the adhesive binds them together and facilitates load transfer between the fibers [8]. Currently, the most commonly used high-strength, high-modulus organic fibers in ballistic materials include aramid fibers (primarily poly-para-phenylene terephthamide, Kevlar), ultra-high molecular weight polyethylene (UHMWPE) fibers, (poly p-phenylene benzobisoxazole) (PBO) fibers, carbon fibers, and glass fibers. These fibers are commonly applied in forms such as fabrics or unidirectional (UD) laminates in the design of personal protective composites, where they exhibit significant energy dissipation effects when subjected to ballistic impacts [9,10,11,12,13,14].

Adhesives play a critical role in ballistic materials. Beyond encapsulating and binding fibers to enable efficient load transfer, they also absorb and dissipate the kinetic energy of projectiles through delamination and peeling damage between fiber layers. Moreover, adhesives help mitigate the transverse deformation of composites caused by high-velocity impacts, thereby reducing the risk of non-penetrating injuries and ultimately minimizing the harm bullets inflict on the human body [15,16]. Mia et al. [17] studied polyurethane/Kevlar fiber composites and found that polyurethane enhanced the friction between Kevlar fiber yarns. The resulting ballistic composite materials exhibited the advantages of low cost, light weight, high hardness, and excellent ballistic performance. Firouzi and Ahmad et al. [18,19] compared the properties of UHMWPE fiber-reinforced ballistic composites with adhesives and those of pure UHMWPE fiber fabrics. Their findings revealed that the addition of adhesives improved both the mechanical and ballistic properties of UHMWPE fibers. Yan et al. [20] investigated the properties of epoxy/UHMWPE composites and vinyl ester/UHMWPE composites. They found that while the deformation modes of the two composites were similar, the vinyl ester/UHMWPE composite, with an adhesive modulus more closely matching that of the fibers, exhibited superior impact resistance. Wang et al. [16] compared the ballistic performance of polyurethane-based composites and composites using three different epoxy resins as matrices. Their results showed that polyurethane-based composites demonstrated better energy absorption and ballistic performance. Han et al. [21] designed and prepared polyurethane adhesives with varying hard segment contents as matrices and studied the ballistic performance and mechanisms of aramid fiber fabric-reinforced polyurethane composites. The results indicated that a soft and tough matrix with a lower hard segment content helped reduce delamination damage by forcing more fibers to interlock, thereby enhancing energy absorption. The prepared aramid fiber fabric-reinforced composites exhibited a relatively low modulus, allowing the matrix to deform together with the fibers during penetration, thus, improving ballistic performance.

Ultra-high molecular weight polyethylene (UHMWPE) fibers are widely regarded as the most efficient organic fibers for ballistic protection materials due to their exceptional properties. With a density lower than water, a specific strength over ten times that of steel wires with the same cross-section, and an impact energy absorption capacity nearly twice that of Kevlar fibers, UHMWPE fibers also possess a stress wave propagation speed that is twice as fast as Kevlar, making them highly effective in dissipating energy during high-velocity impacts. To further enhance their performance in fiber-reinforced adhesive ballistic composites (FRPC), the selection of adhesives must satisfy four key criteria: strong bonding strength with UHMWPE fibers to ensure structural stability, excellent environmental adaptability to suit various application scenarios, a curing or melting temperature below 120 °C to avoid fiber damage, and a post-curing modulus closely matching that of UHMWPE fibers to minimize the risk of interface delamination under stress. Water-based polyurethane adhesive emulsions are commonly used to meet these requirements. In the FRPC fabrication process, UHMWPE fiber bundles are uniformly aligned, impregnated with the adhesive, and dried to form unidirectional (UD) fabrics, which are then laminated in a 0°/90° configuration to create two-layer UD (2UD) sheets. Finally, these sheets are stacked and hot-pressed in a preheated vulcanization press to produce lightweight, durable, and high-performance FRPC panels [22], making them ideal for modern ballistic protection applications.

Water-based polyurethane (WPU) is a resin material formed through the additional reaction of polyisocyanates and polyols [23], characterized by a typical block copolymer structure [24,25]. This structure comprises hard segments, formed by polyisocyanates and chain extenders, which provide strength, stiffness, and chemical stability, as well as soft segments, derived from polyols, which endow the material with excellent toughness, elasticity, and low-temperature resistance. By adjusting the hard segment (HS) content, polyurethane can exhibit a wide range of desirable mechanical properties, such as high elasticity, strength, stiffness, and toughness. Due to its controllable molecular structure, WPU can bridge the performance gaps between other resin adhesives [26,27,28]. Reported polyurethane adhesives have achieved a maximum tensile strength of up to 94.6 MPa [29]. Consequently, environmentally friendly WPU, with tunable thermal and mechanical properties, has been widely used in UHMWPE fiber-based fiber-reinforced polymer composites (FRPC) for ballistic protection applications. However, the relationship between adhesive mechanical properties and the ballistic performance of FRPCs remains poorly understood.

In this study, water-based polyurethane (WPU) was synthesized using polycarbonate (1,6-hexanediol) diol (PCD), isophorone diisocyanate (IPDI), 1,4-cyclohexanedimethanol (CHDM), and 2,2-dimethylolpropionic acid (DMPA) as raw materials via the prepolymer method. WPUs with varying hard segment (HS) contents (35, 45, 55, and 65 wt%) were prepared by adjusting the proportions of PCD, IPDI, CHDM, and DMPA. In this system, PCD serves as the soft segment (SS), while IPDI, CHDM, and DMPA constitute the hard segment (HS). By regulating the HS content, the thermal and mechanical properties of the WPU were controlled. The synthesized WPUs were then used as adhesives to bond ultra-high molecular weight polyethylene (UHMWPE) fibers into unidirectional (UD) fabrics, referred to as single-layered fiber arrangements without interwoven structures. These UD fabrics were subsequently laminated to fabricate fiber-reinforced ballistic composites. Using scanning electron microscopy (SEM), quasi-static mechanical testing, and ballistic experiments, the relationship between the mechanical properties of WPU and the ballistic performance of the composite materials was systematically investigated.

## 2. Materials and Methods

### 2.1. Materials

Polycarbonate (1,6-hexanediol) diol (PCD) with a molecular weight of 1000 was supplied by Yuanli Chemical Group Co., Ltd. (Weifang, China). Isophorone diisocyanate (IPDI, Mn = 222.28) was provided by Covestro Polymers (China) Co., Ltd. (Shanghai, China). We obtained 1,4-Cyclohexanedimethanol (CHDM) from SK Chemicals Co., Ltd. (Shanghai, China), and 2,2-Dimethylolpropionic acid (DMPA) was supplied by Jiangxi Jiyu New Material Co., Ltd. (Fuzhou, Jiangxi, China). Ultra-high molecular weight polyethylene (UHMWPE) fibers were purchased from Jiangsu Weifute New Material Co., Ltd. (Changzhou, China).

### 2.2. Methods

#### 2.2.1. Preparation of WPU

**Figure 1:**
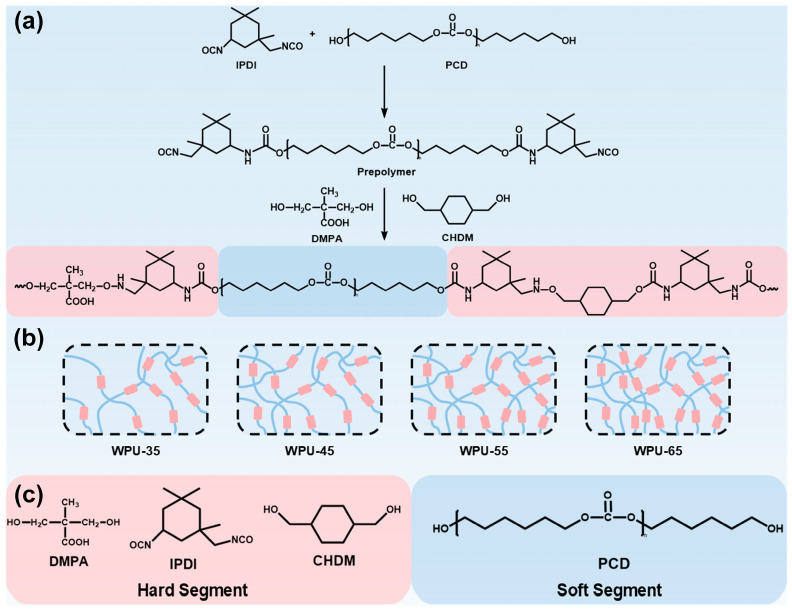
(**a**) Synthetic route and molecular structure of WPU-HS. (**b**) Schematic molecular structures with different hard segment contents: WPU-35, WPU-45, WPU-55, and WPU-65. (**c**) Composition of hard and soft segments, where the blue area represents the soft segment, and the pink area represents the hard segment.

In this study, water-based polyurethanes (WPU) with hard segment (HS) contents of 35%, 45%, 55%, and 65% were synthesized using the prepolymer method [30] by varying the proportions of PCD, IPDI, CHDM, and DMPA. The DA contents of these WPUs were 4.32%, 6.33%, 5.02%, and 7.39%, respectively. DA content represents the proportion of DMPA incorporated into the WPU, which is used to regulate the content of hydrophilic groups (-COOH, carboxyl groups) in the molecular structure. A higher DA content typically enhances emulsion stability and water dispersibility. To prepare the prepolymer, the measured amounts of soft segment (PCD) and hard segment (IPDI) were added to a three-necked flask equipped with a mechanical stirrer. The mixture was reacted at 80 °C with a stirring speed of 120 rpm for 1 h to form an isocyanate-terminated prepolymer. Then, the measured amounts of chain extenders (CHDM and DMPA) were added to the prepolymer mixture and reacted at 80 °C for 4 h to ultimately obtain WPU-HS (wt%). The structure and synthesis steps of WPU are illustrated in Figure 1. The hard segment (HS) content was calculated using the following formula [28,31]:

(1) $$HS(wt\%)=\frac{m_{IPDI}+m_{CHDM}+m_{DMPA}}{m_{PCD}+m_{IPDI}+m_{CHDM}+m_{DMPA}}\times 100\%$$

In this formula, HS (wt%) refers to the mass fraction of the hard segment (HS) in the total mass of the water-based polyurethane (WPU). Here, IPDI, CHDM, and DMPA represent the hard segment, while PCD represents the soft segment. The WPU samples were named as WPU-HS (wt%).

#### 2.2.2. Molecular Dynamics Simulation of WPU-HS

**Figure 2:**
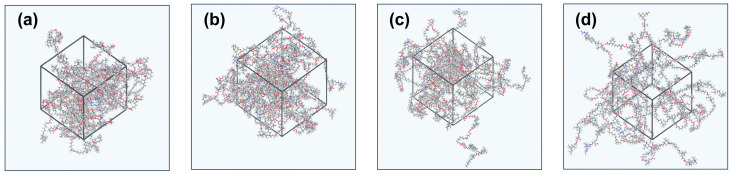
Water-based polyurethane models with different hard segment contents: (**a**) WPU-35. (**b**) WPU-45. (**c**) WPU-55. (**d**) WPU-65.

Molecular dynamics simulation (MS) technology has become a new method in material research due to its efficiency, practicality, versatility, and stability [32,33]. First, the molecular structure drawing tool in the Material Visualizer module of the Materials Studio 2020 MD simulation software was used to construct molecular chain structures of different water-based polyurethane (WPU) systems based on the ratios of PCD, IPDI, CHDM, and DMPA in four types of WPU with varying hard segment contents. Then, the Amorphous Cell Tools module was used to synthesize the WPU unit cell model. The temperature was set to 298 K, the pressure to atmospheric pressure (0.0001 GPa), and the initial relative density to 1.2 g/cm^3^. The Geometry Optimization (GEO) module was applied to optimize the molecular unit cell structure. The resulting WPU model is shown in Figure 2.

#### 2.2.3. Characterization of WPU-HS

The molecular weight information of WPU was obtained using a Waters liquid chromatography system (Waters Corporation, Milford, MA, USA) with a THF-based system. The structure of WPU was analyzed using Fourier transform infrared spectroscopy (FTIR) with a PerkinElmer Spotlight infrared spectrometer (PerkinElmer, Inc., Waltham, MA, USA). The spectrum was scanned 40 times in the range of 4000–400 cm^−1^. The glass transition temperature (Tg) of WPU was analyzed using a Mettler Toledo TGA/DSC1 (Mettler-Toledo International Inc., Columbus, OH, USA) differential scanning calorimeter. The test was conducted in a nitrogen atmosphere with a heating rate of 15 °C/min. The temperature was first increased from room temperature to 150 °C and then cooled to eliminate the thermal history of the material. Subsequently, the sample was heated from −50 °C to 120 °C to obtain DSC data. Dynamic mechanical analysis (DMA) of WPU was performed using a TA Q800 (TA, New Castle, DE, USA) dynamic mechanical analyzer in tensile mode, with a heating rate of 3 °C/min and a frequency of 1 Hz. The temperature range for the measurement was −80 °C to 150 °C, and the storage modulus and loss factor (tan δ) were obtained. The tensile properties of WPU were measured using an Instron universal testing machine (Instron Corporation, Norwood, MA, USA) at a tensile rate of 500 mm/min.

#### 2.2.4. Preparation of WPU-HS/UHMWPE Composites

**Figure 3:**
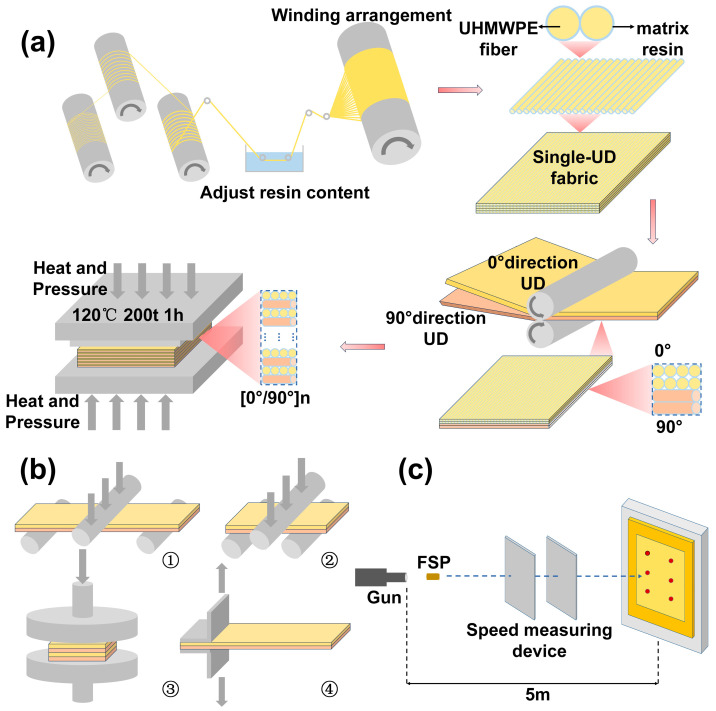
(**a**) Preparation flowchart of WPU-HS/UHMWPE bulletproof composites. (**b**) Schematic diagram of quasi-static mechanical tests, including bending test, ILSS test, compression test, and DCB test. (**c**) Schematic diagram of the ballistic testing device.

As shown in Figure 3a, ultra-high molecular weight polyethylene (UHMWPE) fiber bundles are evenly spread and impregnated into the prepared waterborne polyurethane (WPU). Subsequently, they are wound, arranged, dried, and shaped to form unidirectional (UD) non-woven fabric, where the fibers are aligned in a single direction without interweaving to enhance strength along that direction. The 0°/90° orthogonal composite lamination of two UD layers produces a 2UD sheet. Finally, a certain number of 2UD sheets are laid flat in a preheated vulcanizing hot press and processed at 120 °C and 20 MPa for 20 min. After cooling, WPU-HS (wt%)/UHMWPE composite laminates are obtained. Laminates with different hard segment contents are named WPU-35/UHMWPE, WPU-45/UHMWPE, WPU-55/UHMWPE, and WPU-65/UHMWPE.

#### 2.2.5. Characterization of Quasi-Static Mechanical Properties of WPU-HS/UHMWPE Composites

The samples for the quasi-static mechanical performance tests were cut from the bulletproof composite material using waterjet cutting. The tests were conducted using a universal testing machine, as shown in Figure 3b [34], which includes four test methods: ① bending test, ② interlaminar shear strength (ILSS) test, ③ compression test, and ④ double cantilever beam (DCB) test. As shown in Figure 3b ①, the bending test specimens had dimensions of 154 mm × 13 mm × 4 mm, and the flexural strength and modulus were determined in accordance with the ASTM D7264 standard [35]. The loading span during the test was set to 128 mm, and the crosshead loading rate was 10 mm·min^−1^. The flexural stress was calculated using the following formula:

(2) $$Flexural\;strenth=\frac{3PL}{2bd^{2}}$$

where P is the load (N), L is the span length (mm), b is the specimen width (mm), and d is the specimen thickness (mm). The flexural strain was calculated using the following formula:

(3) $$Flexural\;strain=\frac{6Dd}{L^{2}}$$

where D is the crosshead displacement (mm). Figure 3b ② illustrates the short-beam interlaminar shear test, the specimen dimensions were 24 mm × 8 mm × 4 mm, and the interlaminar shear strength (ILSS) was determined following the ASTM D2344 standard [36]. The loading span was set to 16 mm, and the crosshead loading rate was 1 mm·min^−1^. ILSS was calculated using the following formula:

(4) $$ILSS=\frac{3P_{m}}{4bd}$$

where P_m_ is the maximum load (N). As shown in Figure 3b ③, the quasi-static compression test specimens measured 40 mm × 40 mm × 4 mm. These tests evaluated the load–displacement response of the composite laminates under a compressive load of 50 KN, from which the compressive strength was determined, and the test loading rate was 2 mm·min^−1^. The fourth picture in Figure 3b is the double cantilever beam (DCB) test. The interlaminar Mode I fracture toughness (G_IC_) of the PU-HS/UHMWPE ballistic composite was measured using a DCB test in accordance with the ASTM D5528 standard [37]. The specimens had dimensions of 160 mm × 25 mm × 4 mm, with a 65 mm × 25 mm PTFE (polytetrafluoroethylene) film inserted at the mid-plane during fabrication to serve as the initial crack, ensuring a sharp crack tip and uniform propagation. To facilitate tensile loading, two piano hinges were bonded to the front end of each specimen using epoxy resin (AB adhesive), with the loading point located 50 mm from the end of the initial crack. The test was conducted on a universal testing machine at a constant loading rate of 5 mm/min. During the test, the crack propagation was monitored visually using a traveling microscope or by marking the crack front, while the applied load and displacement were recorded continuously. The fracture toughness G_IC_ was calculated using the following formula:

(5) $$G_{IC}=\frac{3P\delta }{2ba}$$

where P is the load (N), δ is the displacement (mm), b is the specimen width (mm), and a is the crack length (mm). The crack length a was determined as the sum of the initial crack length (a0) and the crack propagation length (Δa).

#### 2.2.6. Ballistic Performance Testing of WPU-HS/UHMWPE Composites

According to the NATO STANAG 2920 standard [38], the ballistic testing device as shown in Figure 3c, the ballistic impact setup consists of a 7.62 mm caliber ballistic gun, a velocity measurement device, fragment-simulating projectiles (FSP), and composite material samples. The FSP is made of cast alloy steel, weighing 1.1 g with a diameter of 5.5 mm. The FSP is fired at a composite laminate positioned 5 m away from the gun muzzle. The initial velocity of the FSP is set within the range of 460 m/s to 580 m/s. The V50 value is determined as the average velocity at which, within a specified number of shots, half of the projectiles completely penetrate the laminate, and the other half do not. The kinetic energy dissipated by the laminate during projectile penetration was calculated as the energy absorbed by the laminate, which is equal to the initial energy of the projectile minus the residual energy after penetrating the composite material. The absorbed energy (ΔE) was determined by subtracting the residual energy of the projectile after penetration from its initial energy [39,40]. The calculation formula is as follows:

(6) $$\Delta E=\frac{1}{2}m\left(V_{i}^{2}-V_{r}^{2}\right)$$

where ΔE is the energy absorbed by the composite laminate, m (kg) is the mass of the simulated projectile, Vᵢ is the initial velocity of the projectile (m/s), and Vᵣ is the residual velocity of the projectile after penetration (m/s). The specific energy absorption (SEA) value represents the energy absorption efficiency of the laminate per unit areal density. SEA was calculated using the following formula:

(7) $$SEA=\frac{\Delta E}{\sigma }$$

where σ (kg/m^2^) is the areal density of the composite laminate.

#### 2.2.7. Morphological Analysis of WPU-HS/UHMWPE Composites

Before ballistic testing, the surfaces of the four composites were examined and gold-coated to analyze the bonding between fibers and the adhesive using a scanning electron microscope (SEM). After the ballistic tests, the surface morphology and SEM images were used to observe the deformation and failure modes of the fibers after projectile penetration.

## 3. Results and Discussion

### 3.1. Molecular Dynamics Simulation Results of WPU-HS

To verify whether the constructed water-based polyurethane (WPU) model aligns with actual physical and chemical behaviors, molecular dynamics simulations were performed using the Dynamics function in the Forcite module. The simulations employed an NPT ensemble, with constant molecular weight, pressure, and temperature parameters. The temperature was set to 298 K, and the system underwent 20,000 iterations for energy minimization, with a simulation duration of 20 ps. These conditions ensured the reliability and validity of the WPU model. Subsequently, the Cohesive Energy Density (CED) function in the Forcite module was utilized to analyze the cohesive energy density of the model. CED is defined as the energy per unit volume required to overcome intermolecular forces within a material. It quantitatively reflects the degree of intermolecular bonding within the structure, representing the energy required to transform one mole of a polymer from the liquid phase to the gaseous phase under isothermal conditions. Hence, CED is a critical physical parameter that characterizes the strength of intermolecular interactions and serves as an indicator of the material’s thermodynamic stability. The CED values of water-based polyurethanes (WPU) with different hard segment contents are shown in Table 1.

As illustrated in Figure 2, the WPU simulation model with a hard segment content of 35% exhibits highly flexible molecular chains, leading to a more entangled arrangement of molecular chains within the unit cell. In contrast, when the hard segment content reaches 65%, the molecular chains exhibit significantly higher rigidity, resulting in straighter and stiffer chain conformations within the unit cell model. At this stage, the excessive rigidity of the material leads to a marked reduction in flexibility and elasticity, causing a significant decrease in elongation at break. Consequently, the material becomes brittle and is more susceptible to fracture under the impact or tensile stress. When the CED is within the range of 300 J/cm^3^ to 400 J/cm^3^, the intermolecular interactions of the polymer are moderate. It is noteworthy that the CED values of WPUs with varying hard segment contents all fall within this range. Under such conditions, the polymer achieves an optimal balance between flexibility and rigidity, providing the material with both excellent toughness and sufficient mechanical strength. Specifically, the molecular dynamics simulation results show that the CED values of WPU-35, WPU-45, WPU-55, and WPU-65 are 351.7 J/cm^3^, 330.7 J/cm^3^, 319.8 J/cm^3^, and 354.4 J/cm^3^, respectively. Among these, both WPU-35 and WPU-65 exhibit relatively high CED values, yet the reasons for this are different. The high CED of WPU-35 is primarily attributed to the high concentration of carbonate groups in the soft segment, which enhances molecular chain flexibility and increases intermolecular interactions. In contrast, the high CED of WPU-65 is mainly due to the increased rigidity of its molecular chains and enhanced hydrogen bonding interactions, which significantly contribute to the overall cohesion. Meanwhile, the lower CED values of WPU-45 and WPU-55 are instead likely due to increased molecular chain flexibility or reduced intermolecular interactions. This variation in CED, driven by structural differences, highlights the ability of the polymer to achieve a balance between toughness and mechanical strength by fine-tuning its molecular architecture.

### 3.2. Chemical Structure, Thermal, and Mechanical Properties of WPU-HS

#### 3.2.1. Molecular Weight Testing of WPU-HS

The molecular weight of polyurethane reflects the length of its molecular chains or the average number of repeating units, which directly affects its properties [41,42]. The longer the polyurethane molecular chains and the higher their molecular weight, the stronger the intermolecular interactions, which typically result in better mechanical properties and film-forming abilities. However, excessively high molecular weight may lead to an increase in emulsion viscosity, negatively affecting stability and processability. The number-average molecular weight (M_n_) is the arithmetic mean of molecular weights and reflects the average chain length of the polymer. A higher M_n_ indicates longer polyurethane molecular chains, which contribute to improved strength, toughness, and durability. The weight-average molecular weight (M_w_) is a molecular weight average weighted by chain length, making it more sensitive to high-molecular-weight chains and reflecting the contribution of larger chains to the overall performance. The molar-mass dispersity (Đ_M_) is the ratio of M_w_ to M_n_, representing the breadth of molecular weight distribution [43]. A larger Đ_M_ indicates a wider molecular weight distribution, reflecting less uniformity in the polymer chains. The molecular weight data of the synthesized WPUs are summarized in Table 1.

At lower hard segment content (e.g., WPU-35), the higher proportion of soft segments increases molecular chain flexibility, facilitating chain growth during polymerization. This results in a moderate molecular weight (M_n_ = 1.22 × 10^4^, M_w_ = 3.74 × 10^4^) and a larger Đ_M_ (3.06), as insufficient hard segment content limits chain alignment and broadens the molecular weight distribution. In contrast, at higher hard segment content (e.g., WPU-65), strong interactions between hard segments, such as hydrogen bonding and microphase separation, hinder chain growth. This leads to a lower molecular weight (M_n_ = 0.74 × 10^4^, M_w_ = 2.54 × 10^4^) and the highest Đ_M_ (3.42), indicating reduced compatibility between soft and hard segments and uneven chain growth. For WPU-45 and WPU-55, the balanced ratio of soft to hard segments promotes more uniform chain growth, resulting in higher molecular weights (M_n_ = 1.67 × 10^4^, M_w_ = 4.81 × 10^4^ for WPU-45; M_n_ = 1.17 × 10^4^, M_w_ = 2.81 × 10^4^ for WPU-55) and the lowest Đ_M_ values (2.89 and 2.39, respectively). The lower Đ_M_ reflects more uniform molecular chain growth due to balanced segment interactions, leading to a more ordered molecular weight distribution.

#### 3.2.2. Fourier Transform Infrared Spectroscopy (FTIR) Analysis of WPU-HS

**Figure 4:**
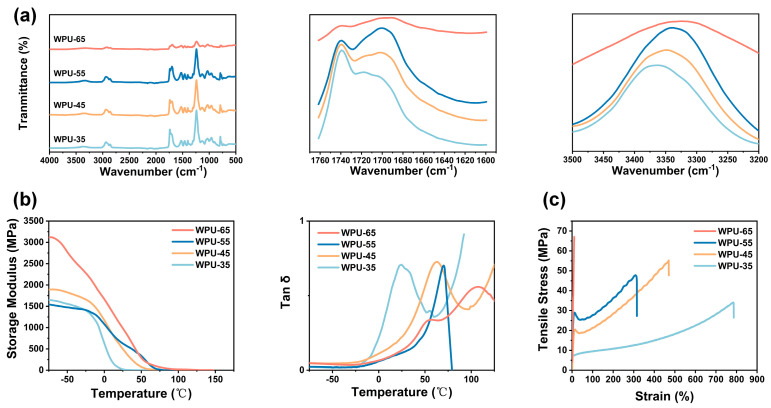
Structural, thermal, and mechanical performance analysis of WPU-HS: (**a**) Fourier transform infrared spectroscopy (FTIR) analysis of WPU-HS. (**b**) Dynamic mechanical analysis (DMA) of WPU-HS, showing storage modulus and tanδ. (**c**) Tensile properties of WPU-HS.

Fourier transform infrared spectroscopy (FTIR) is a powerful analytical technique for identifying the chemical composition and functional groups of polymers by detecting their characteristic absorption peaks. The FTIR spectrum of water-based polyurethane (WPU-HS) is shown in Figure 4a. The absence of the isocyanate group (-NCO) peak at 2250 cm^−1^ indicates the complete reaction of isocyanate during the synthesis process. Furthermore, the presence of characteristic absorption peaks confirms the successful synthesis of polyurethane. These include the C-O-C stretching vibrations around 1000 cm^−1^, the carbonyl group (-C=O) stretching vibrations within the range of 1680–1750 cm^−1^, and the urethane bond (-NHCOO-) with –NH stretching vibrations at 3300–3400 cm^−1^ [31,44,45]. For the carbonyl group (-C=O), the observed absorption band within 1680–1750 cm^−1^ can be attributed to the stretching vibrations of the urethane groups. Specifically, this band often exhibits a shoulder due to the coexistence of hydrogen-bonded carbonyl groups (1700 cm^−1^) and free carbonyl groups (1730 cm^−1^). As the hard segment (HS) content increases, the intensity of the hydrogen-bonded carbonyl peak (1700 cm^−1^) becomes more pronounced, while the free carbonyl peak (1730 cm^−1^) diminishes. This trend indicates an increase in hydrogen bonding interactions, which is a direct result of the higher proportion of hard segments in the polymer structure. Additionally, the hydrogen-bonded -NH stretching peak at 3300–3350 cm^−1^ shifts to lower frequencies as the HS content increases. This shift suggests stronger hydrogen bonding interactions due to the increased density of hard segment domains [46]. Such changes in the FTIR spectrum provide critical evidence for the structural evolution of the polymer, particularly the role of hard segment content in influencing molecular interactions and phase separation within WPU-HS.

#### 3.2.3. Differential Scanning Calorimetry (DSC) Analysis of WPU-HS

DSC testing is an important tool for studying the thermal properties of polyurethane [47,48]. Table 2 lists the glass transition temperatures (T_g_^DSC^) of WPU-HS. The T_g_^DSC^ of WPU-HS results from the combined effects of the hard and soft segments. As the hard segment content increases, the T_g_^DSC^ also increases. This is because rigid molecular chains require more energy (higher temperatures) to overcome intermolecular constraints and undergo the glass transition. Moreover, the hard segments contain highly polar groups (-NH, C=O), which readily form numerous hydrogen bonds. These hydrogen bonds enhance the intermolecular interactions, leading to the formation of more physical crosslinking points within the hard segments [49], thereby making molecular motion more difficult.

#### 3.2.4. Dynamic Mechanical Analysis (DMA) of WPU-HS

DMA testing provides information about the storage modulus and loss factor (tanδ) of polyurethane at different temperatures, and it can accurately determine the glass transition temperature (T_g_^DMA^). As shown in Figure 4b and Table 2, the T_g_^DMA^ values of WPU-35, WPU-45, WPU-55, and WPU-65 are 23.05 °C, 62.81 °C, 70.23 °C, and 106.65 °C, respectively, which correspond to the T_g_^DSC^ values of WPU [21]. At low temperatures, the molecular motion of WPU chains is restricted, and the flexibility of the molecular chains is significantly reduced, resulting in a frozen state. This leads to higher rigidity and elastic energy storage capacity, thereby exhibiting a higher storage modulus. As the temperature increases, the frozen main chains of the molecular structure begin to move, and this motion weakens the intermolecular interactions, reducing the energy storage capacity of WPU. The hard segment domains in WPU are the aggregation regions of hard segments within the polymer network. With an increase in hard segment content, the size and quantity of hard segment domains also increase, enhancing the degree of crosslinking, chain rigidity, and deformation resistance of the molecular structure. Consequently, the polyurethane can store elastic energy more effectively when subjected to external forces, resulting in a higher storage modulus. The tanδ represents the ratio of the loss modulus to the storage modulus. When the storage modulus exceeds the loss modulus, tanδ is less than 1. For WPU with varying hard segment contents, tanδ is consistently less than 1, indicating that WPU stores energy primarily in an elastic form.

#### 3.2.5. Mechanical Property Analysis of WPU-HS

The tensile properties of WPU are shown in Figure 4c and Table 2. The tensile strengths of WPU-35, WPU-45, WPU-55, and WPU-65 are 34.11 MPa, 55.22 MPa, 43.09 MPa, and 67.05 MPa, respectively. The tensile strength and Young’s modulus of WPU-HS increase with the rising hard segment content, while the elongation at break decreases sharply from 786.63% for WPU-35 to 10.55% for WPU-65. As the hard segment content increases, the number of hard segment domains and physical crosslinking points also grows, resulting in higher stiffness for WPU, which enhances its tensile strength and Young’s modulus. During the tensile process, the soft segments can undergo significant deformation, absorbing part of the stress. However, the presence of hard segment domains limits this deformation process. When the hard segment content is high, the hard segment domains not only restrict the deformation of the soft segments but also lead to a decline in the overall elasticity and ductility of the polyurethane. Ultimately, this reduces the elongation at break of the polyurethane.

### 3.3. Quasi-Static Mechanical Properties of WPU-HS/UHMWPE Composites

The quasi-static properties of the composite materials are closely related to their ballistic performance. Improvements in quasi-static properties can enhance the structural strength, stiffness, and toughness of the composites under low-speed and static loading, which provides a foundation for their ballistic performance.

#### 3.3.1. Quasi-Static Flexural Properties of WPU-HS/UHMWPE Composites

**Figure 5:**
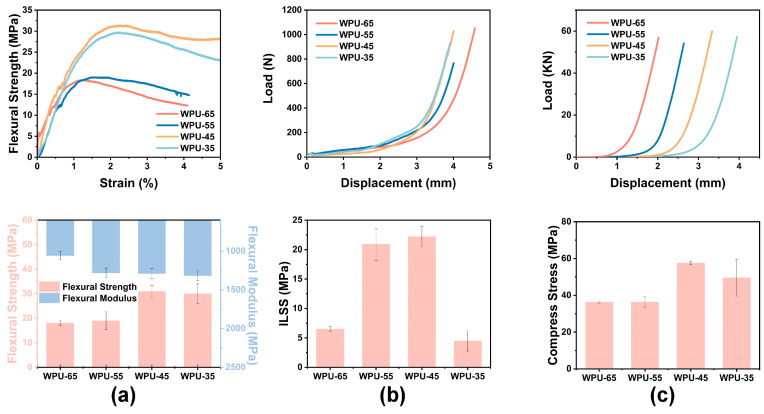
Quasi-static mechanical properties of WPU-HS/UHMWPE composites: (**a**) Flexural stress–strain curves and flexural modulus of WPU-HS/UHMWPE composites. (**b**) Interlaminar shear stress–strain curves and ILSS of WPU-HS/UHMWPE composites. (**c**) Compressive load–displacement curves and compressive strength of WPU-HS/UHMWPE composites.

Bullet impacts induce bending deformation in composite materials, which further promotes interlayer shear and delamination damage. Superior flexural properties enable the material to absorb impact energy through deformation, thereby reducing the direct transmission of shock waves [50]. High flexural strength indicates that the material possesses excellent strength, toughness, and interlayer bonding, ensuring it can effectively distribute stress and avoid failure under external loads or impacts. Therefore, flexural performance testing can indirectly reflect certain aspects of ballistic performance, particularly when evaluating the overall mechanical behavior and impact resistance of composite materials, where this correlation is especially significant. As shown in Figure 5a, the stress–strain curve of WPU-45/UHMWPE exhibits the highest slope, corresponding to its maximum flexural strength and flexural modulus. This can be attributed to the lower hard segment content in the WPU matrix, which provides better flexibility, allowing the load to be quickly and effectively transferred to the fibers, thereby promoting fiber deformation and enhancing flexural performance. Additionally, WPU-45/UHMWPE exhibits yielding behavior during the test, which is caused by compression failure in the upper layers of the laminate, leading to a decrease in strength, followed by strength recovery due to tensile deformation of the fibers in the lower layers [21]. In contrast, WPU-35/UHMWPE, WPU-55/UHMWPE, and WPU-65/UHMWPE may experience uneven load transfer, resulting in weaker tensile deformation of the bottom fibers and, thus, no significant strength recovery.

#### 3.3.2. Interlaminar Shear Strength of WPU-HS/UHMWPE Composites

When composite materials are subjected to high-velocity projectile impacts, the projectile generates significant stress, leading to localized damage in the material. The interlaminar shear strength (ILSS) refers to the composite material’s ability to resist interlayer delamination when subjected to shear loads between different layers. Composite materials are typically composed of stacked fiber layers, and the strength between these layers determines the material’s resistance to damage under external forces. Higher ILSS enables the material’s layers to better resist interlayer delamination and separation, preventing failure during impact and thereby effectively improving ballistic performance [51,52,53]. Figure 5b shows the short-beam shear load–displacement curves and ILSS of WPU-HS/UHMWPE composite laminates. The incorporation of WPU enhances the stiffness of the laminate, leading to a rapid increase in load. However, a higher hard segment content increases the brittleness of the interlaminar fibers, making them less susceptible to shear damage, while also potentially causing the rigid regions in the material to become overly concentrated. This results in stress concentration at the interfacial contact areas between layers, increasing the risk of fracture or failure and ultimately reducing the ILSS. Conversely, an excessively low hard segment content increases the toughness of the matrix, making the laminates more prone to bending during testing, leading to bending failure and fiber slippage between layers, which also reduces the ILSS. As a result, WPU-45/UHMWPE exhibits the highest ILSS. The manufacturing process of WPU-HS/UHMWPE composite materials has a significant impact on their mechanical properties. If the processing temperature is too low or too high, or if the holding time is insufficient, issues such as fiber layer slippage or adhesive debonding may occur during shear sample or static mechanical testing. These issues can result in reduced mechanical performance of the samples, preventing the true mechanical properties of the composite material from being reflected. The melting point of UHMWPE fibers is 120 °C, When the hot-pressing process is conducted at this temperature, it is incompatible with the properties of high hard-segment WPU. WPU-65 exhibits poor flowability at this temperature and cannot properly bond with the fibers, resulting in reduced quasi-static mechanical properties of the WPU-65/UHMWPE composite material.

#### 3.3.3. Compressive Strength of WPU-HS/UHMWPE Composites

Under the high-speed impact of a bullet, composite materials are subjected to compressive waves along the thickness direction [54], resulting in compression damage. Higher axial compressive strength generally contributes to improving the ballistic resistance of composite materials, as it enhances material stiffness and interlayer bonding strength, thereby increasing energy absorption capacity and reducing the projectile’s velocity within the composite. As shown in Figure 5c, the higher the hard segment content, the greater the material stiffness, and the more sensitive the load–displacement response. WPU-45/UHMWPE and WPU-55/UHMWPE exhibit higher compressive strength. This is because WPU-35, with its lower hard segment content, has excessive adhesive flexibility, causing the fibers to slip out of position, which reduces compressive strength. On the other hand, WPU-65 has a higher glass transition temperature (T_g_), making molecular chain motion more difficult. The poor flowability and uneven distribution of the adhesive within the fibers further lead to a decrease in compressive strength.

#### 3.3.4. Model-I Interlaminar Fracture Toughness of WPU-HS/UHMWPE Composites

**Figure 6:**
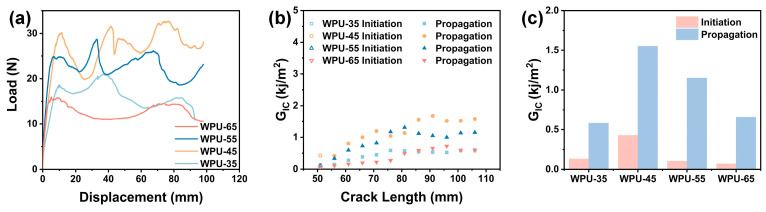
(**a**) Load–displacement curves of WPU-HS/UHMWPE composites. (**b**) R-curves of WPU-HS/UHMWPE composites. (**c**) Average energy release rates for crack initiation (G_IC-ini_) and crack propagation (G_IC-prop_) of WPU-HS/UHMWPE composites.

In FRPC, the fibers are typically responsible for bearing most of the stress, while the adhesive serves to bond the fibers and transfer stress. Under high-speed impact, the bonding interface can fail, leading to debonding between the fibers and the adhesive, and the composite material exhibits delamination damage. Figure 6 shows the load–displacement curves, R-curves, and the average energy release rates for crack initiation (G_IC-ini_) and crack propagation (G_IC-prop_) of WPU-HS/UHMWPE composite materials. The load–displacement curves of WPU-HS/UHMWPE exhibit a wavelike fluctuation, indicating that cracks are continuously propagating during testing and that greater loads are required during crack propagation. The initial crack load–displacement curve of WPU-45/UHMWPE is the highest, suggesting that the excellent toughness of WPU-45 makes crack propagation more difficult [55,56]. The R-curve, also known as the delamination-resistance curve, is an important tool for studying the relationship between composite materials and crack propagation resistance. WPU-45/UHMWPE demonstrates stronger delamination resistance, with the highest energy release rates for both crack initiation (G_IC-ini_) and crack propagation (G_IC-prop_), reaching 0.43 kJ/m^2^ and 1.55 kJ/m^2^, respectively. Moreover, the G_IC-prop_ value shows an upward trend during crack propagation. As the hard segment content increases, both G_IC-ini_ and G_IC-prop_ gradually decrease.

### 3.4. Ballistic Performance of WPU-HS/UHMWPE Composites

**Figure 7:**
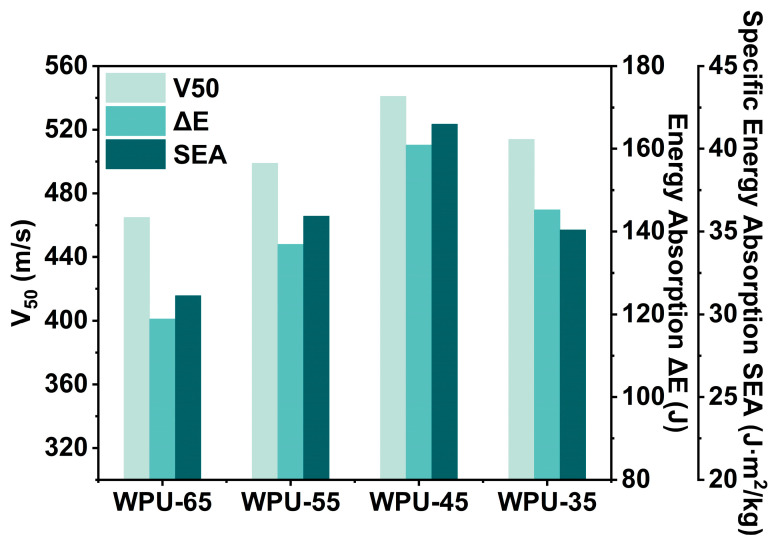
Ballistic performance of WPU-HS/UHMWPE composites: V_50_; energy absorption (ΔE); and specific energy absorption (SEA).

For ballistic composite materials, V50 value, energy absorption (ΔE), and specific energy absorption (SEA) are three key indicators for evaluating ballistic performance, reflecting the material’s capability in actual protective applications. Among them, the V50 value represents the critical velocity at which the material can effectively prevent a bullet from penetrating. Energy absorption describes how much energy the ballistic material can absorb during projectile impact, while SEA refers to the energy absorbed per unit areal density of the material. The V50, ΔE, and SEA values of WPU-HS/UHMWPE composite laminates are shown in Figure 7 and Table 3. For composite laminates with the same number of fiber layers, the V50 values of WPU-65/UHMWPE, WPU-55/UHMWPE, WPU-45/UHMWPE, and WPU-35/UHMWPE are 465, 499, 541, and 514 m/s, respectively, with WPU-65 showing the lowest value. Similarly, the ΔE values are 118.92 J for WPU-65, 136.95 J for WPU-55, 160.97 J for WPU-45, and 145.31 J for WPU-35, with WPU-45 exhibiting the highest energy absorption, an increase of 35.36%, 17.54%, and 10.78% compared to WPU-65, WPU-55, and WPU-35, respectively. As for SEA, WPU-45 also outperforms the others, reaching 41.49 J·m^2^/kg, which is 33.28%, 15.44%, and 18.21% higher than WPU-65, WPU-55, and WPU-35, respectively. These results suggest that an excessively high hard segment content, as in WPU-65, leads to a rigid and brittle matrix with high hardness and modulus, which may hinder fiber deformation and reduce the material’s ability to absorb and dissipate energy. Consequently, high hardness and modulus in the matrix do not always enhance ballistic performance. In contrast, WPU-45 demonstrates excellent ballistic performance with a thinner laminate thickness, highlighting its superior mechanical properties that significantly improve the protective capabilities of the composite material.

### 3.5. Microscopic Morphology Analysis of WPU-HS/UHMWPE Composites

**Figure 8:**
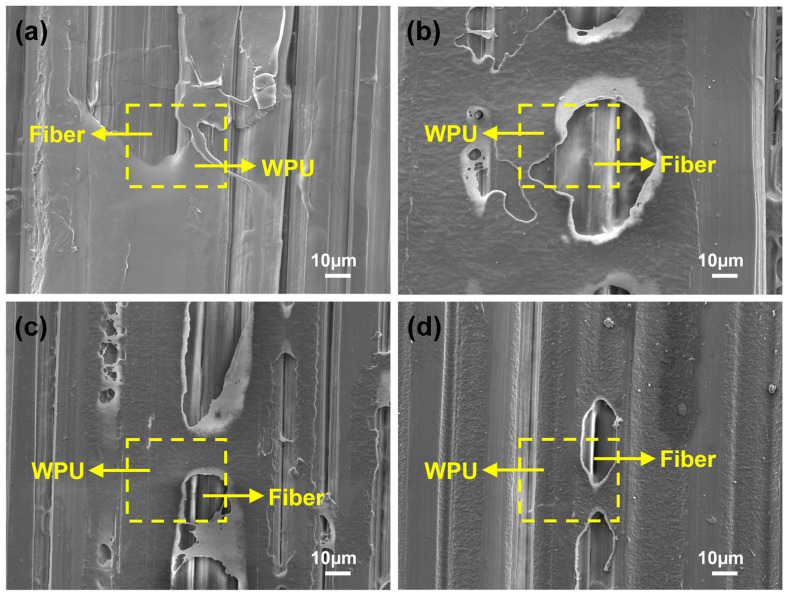
Surface morphology of UHMWPE fibers combined with WPU: (**a**) WPU-35/UHMWPE. (**b**) WPU-45/UHMWPE. (**c**) WPU-55/UHMWPE. (**d**) WPU-65/UHMWPE.

Figure 8 presents the SEM images of UHMWPE fiber surfaces bonded with WPU containing four different hard segment contents. The UHMWPE unidirectional fabric is effectively bonded by WPU, with all four types of WPU forming a uniform and complete coating over the fiber surfaces. The UD fabric maintains a well-aligned structure without noticeable gaps. During projectile impact, all four ballistic composite materials demonstrate the ability to provide effective protection.

**Figure 9:**
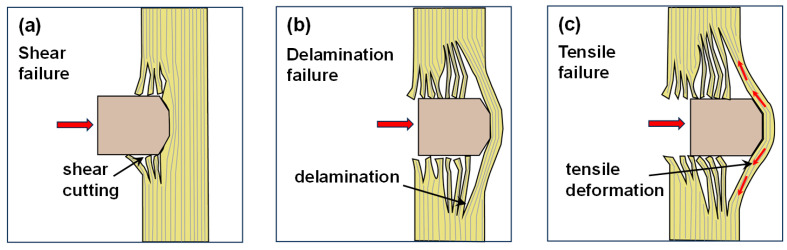
The penetration process of projectile FSP and energy absorption mechanisms: (**a**) Shear failure. (**b**) Delamination failure. (**c**) Tensile failure. The red arrows in the figure indicate the bullet impact.

**Figure 10:**
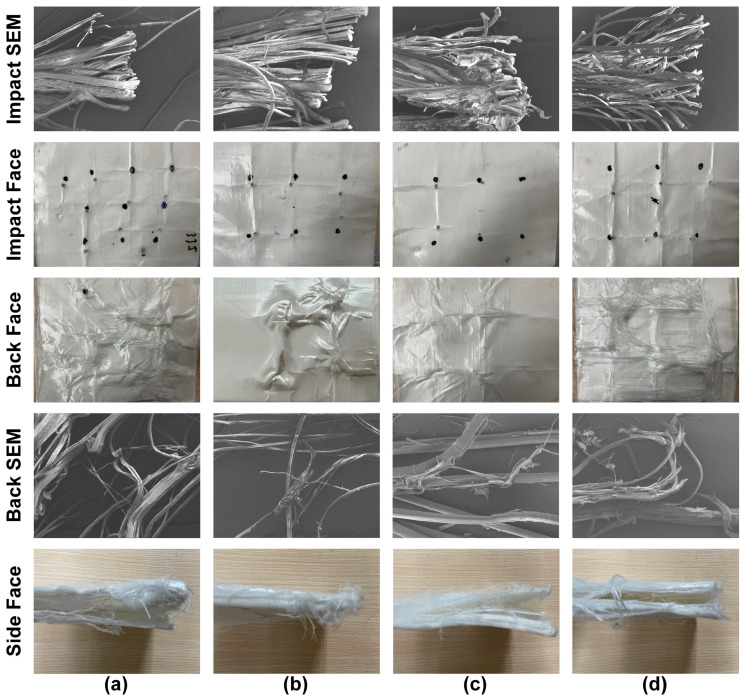
Surface morphology and SEM micrographs of impact, back, and side faces of WPU-HS/UHMWPE composites: (**a**) WPU-35/UHMWPE. (**b**) WPU-45/UHMWPE. (**c**) WPU-55/UHMWPE. (**d**) WPU-65/UHMWPE.

Adhesives play a critical role in ballistic composite materials, making it essential to understand the deformation and failure mechanisms during ballistic impact. Such an understanding is crucial for optimizing the design of both the composite materials and the molecular structure of adhesives. Figure 9 illustrates the penetration process of the projectile FSP and the energy absorption mechanisms. Figure 10 shows the different damage characteristics exhibited on the impact face and back face of the WPU-HS/UHMWPE ballistic composite material after ballistic testing. The ballistic testing standard used is NATO STANAG 2920, and the specific testing setup is shown in Figure 3c.

As shown in Figure 9, the main energy absorption mechanisms of the composite laminates include the following three failure modes: First, the upper fiber layers undergo shear failure due to shear forces. Second, the middle fiber layers absorb and dissipate energy through delamination failure and crack propagation. Finally, the remaining fiber layers at the bottom experience tensile failure. When the projectile comes into contact with the surface of the composite laminate, compressive stress is generated along the thickness direction of the material. As the ballistic penetration deepens, this compressive stress transitions into shear forces, resulting in shear failure and fiber breakage, as indicated by the impact face and SEM images in Figure 10. Subsequently, the fiber layers in the composite laminate absorb and dissipate the projectile’s energy through delamination failure, as shown in the side face of Figure 10. At this stage, the stress is dispersed, delaying the overall structural failure of the composite laminate. Finally, the bending deformation of the remaining fiber layers in the laminate generates axial bending tension in the fibers, leading to tensile deformation and failure. The damage on the back face is primarily characterized by tensile failure, as shown in the back face and SEM images in Figure 10. The above failure and energy absorption mechanisms were observed in all four groups of composite laminates fabricated with different adhesives in this study. The energy absorption capacity of the WPU-HS/UHMWPE composite materials is closely related to the balance between the hard segment and soft segment in the molecular structure. For instance, in WPU-65/UHMWPE and WPU-55/UHMWPE, a higher hard segment content increases the rigidity of the composite, making shear failure the dominant energy absorption mechanism, as shown in the side face of Figure 10. However, excessive rigidity leads to stress concentration at internal interfaces, reducing energy absorption due to plastic deformation and exacerbating delamination failure. In the case of WPU-35/UHMWPE, delamination is also relatively severe. This is because the lower hard segment content increases the material’s flexibility, which reduces interfacial bonding strength and makes fibers more prone to sliding against the adhesive. Consequently, the proportion of energy absorbed by multi-layer delamination failure increases.

**Figure 11:**
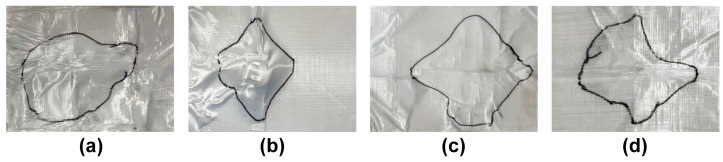
Back face delamination area of WPU-HS/UHMWPE composites: (**a**) WPU-35/UHMWPE. (**b**) WPU-45/UHMWPE. (**c**) WPU-55/UHMWPE. (**d**) WPU-65/UHMWPE.

In addition, Figure 11 shows the back-face delamination areas of individual bullet holes for each sample, and the delamination areas were quantitatively analyzed using Photoshop. The measured delamination areas of WPU-65/UHMWPE, WPU-55/UHMWPE, WPU-45/UHMWPE, and WPU-35/UHMWPE are 104.13 cm^2^, 98.91 cm^2^, 50.79 cm^2^, and 82.93 cm^2^, respectively. The results reveal significant differences in delamination areas corresponding to variations in hard segment content, indicating that the interfacial bonding strength and energy dissipation capacity of the materials are influenced by their composition. Specifically, WPU-65/UHMWPE exhibited the largest delamination area due to its high rigidity and insufficient toughness, resulting in significant delamination and lower energy dissipation efficiency under impact. WPU-55/UHMWPE showed a slightly smaller delamination area, reflecting improved but still suboptimal interfacial bonding performance. WPU-35/UHMWPE, despite having better toughness, showed increased delamination due to insufficient rigidity caused by its low hard segment content. In contrast, WPU-45/UHMWPE exhibited the smallest delamination area, indicating an optimal balance between hard and soft segments, which enabled superior energy absorption and dissipation under ballistic impact.

## 4. Conclusions

This study synthesized waterborne polyurethane (WPU) adhesives with hard segment contents of 35%, 45%, 55%, and 65% (wt%) and systematically investigated the structural and mechanical properties of the adhesives, as well as the mechanical and ballistic performance of their UHMWPE composites. The results indicate that as the hard segment content increases, the thermal and mechanical properties of the WPU adhesive improve significantly, transitioning from soft-segment-dominated flexibility to hard-segment-dominated rigidity and brittleness. Through quasi-static mechanical tests, ballistic impact tests, and SEM morphological analyses of WPU-HS/UHMWPE composites, it was found that the WPU-45 adhesive-based composites exhibited the best overall performance. The WPU-45/UHMWPE composite demonstrated superior bending strength, interlaminar shear strength, compressive strength, and interlaminar fracture toughness compared to other composites. Morphological observations further revealed that the WPU-45/UHMWPE absorbed more kinetic energy from bullets. In contrast, the composite with the highest hard segment content (WPU-65) exhibited the lowest quasi-static mechanical properties. The ballistic performance of WPU-HS/UHMWPE composites showed a fluctuating decline with increasing hard segment content. As the hard segment content decreased, the WPU adhesive transitioned from rigidity and brittleness to softness and toughness, significantly improving the ballistic performance of the composites. This suggests that while high-hardness and high-modulus adhesives provide greater initial strength, they restrict fiber deformation, limiting the energy absorption capacity through fiber deformation and damage. Furthermore, insufficient interlaminar toughness in rigid adhesives leads to severe delamination damage, further reducing the energy absorption efficiency of the fibers. Additional analysis revealed distinct deformation mechanisms and energy dissipation efficiencies for each composite. WPU-45/UHMWPE achieved the best balance between rigidity and toughness, enabling efficient load transfer, controlled delamination, and synergistic deformation with fibers, resulting in the most effective energy absorption and overall ballistic performance. In contrast, softer and tougher adhesives can deform synergistically with fibers during bullet penetration, effectively suppressing interlaminar delamination damage. This allows for greater load transfer and higher kinetic energy absorption. Consequently, the WPU-45 adhesive achieves the optimal balance between hardness, modulus, and toughness, producing composites with thinner thickness, higher areal density, and excellent ballistic performance under the same number of fiber layers.

## Figures and Tables

**Table 1:** CED and molecular weight of WPU.

Samples	CED (J/cm^3^)	Number-Average Molecular Weight (M_n_)	Weight-Average Molecular Weight (M_w_)	Đ_M_
WPU-35	351.7	1.22 × 10^4^	3.74 × 10^4^	3.06
WPU-45	330.7	1.67 × 10^4^	4.81 × 10^4^	2.89
WPU-55	319.8	1.17 × 10^4^	2.81 × 10^4^	2.39
WPU-65	354.4	0.74 × 10^4^	2.54 × 10^4^	3.42

**Table 2:** Thermal and mechanical properties of WPU-HS.

Samples	T_g_^DSC^ (°C)	T_g_^DMA^ (°C)	Storage Modulus (25 °C) (MPa)	Tanδ (Max)	Tensile Strength (MPa)	Strain (%)	Young’s Modulus (MPa)
WPU-35	22.01	23.05	21.65	0.71	34.11	786.63	4.35
WPU-45	57.75	62.81	496.11	0.73	55.22	470.11	11.75
WPU-55	74.50	70.23	626.82	0.71	43.09	239.86	17.97
WPU-65	104.75	106.65	985.93	0.56	67.05	10.55	636.77

**Table 3:** Ballistic performance of WPU-HS/UHMWPE composites.

Samples	Thickness (mm)	Areal Density (kg/m^2^)	V_50_ (m/s)	Energy Absorption (J)	SEA (J·m^2^/kg)
WPU-65	4.47	3.82	465	118.92	31.13
WPU-55	4.33	3.81	499	136.95	35.94
WPU-45	4.27	3.88	541	160.97	41.49
WPU-35	4.46	4.14	514	145.31	35.10

## Data Availability

The original contributions presented in this study are included in the article. Further inquiries can be directed to the corresponding author.
